# Can middle school students counteract academic burnout through physical exercise? A cross-sectional study from China

**DOI:** 10.3389/fpsyg.2026.1829239

**Published:** 2026-07-06

**Authors:** Wen Tao Zhu, Rui Qin, Xin Tan, Xinjuan Zhao

**Affiliations:** 1 School of Football, Chengdu Sport University, Chengdu, China; 2 School of Sports Medicine and Health, Chengdu Sport University, Chengdu, China; 3 School of Physical Education, Sichuan Tourism University, Chengdu, China; 4 School of Physical Education, Leshan Normal University, Leshan, China

**Keywords:** academic burnout, cross-sectional study, emotion regulation, expressive suppression, middle school students, physical exercise

## Abstract

**Objective:**

This study examined the association between physical exercise and academic burnout among Chinese middle school students and tested whether expressive suppression mediated this association.

**Methods:**

A questionnaire survey was conducted using random cluster sampling among middle school students from Guangdong, Sichuan, Zhejiang, Beijing, Henan, and Hainan, China. A total of 3,786 valid responses were obtained. Physical exercise, expressive suppression, and academic burnout were measured using validated scales. Data were analyzed using SPSS 26.0 and AMOS 24.0 for descriptive statistics, correlation analysis, path analysis, and SEM-based mediation testing, with gender, age, and grade controlled as demographic covariates.

**Results:**

(1) Physical exercise was significantly negatively correlated with academic burnout (β = –0.192, *p* < 0.001); higher levels of physical exercise were associated with lower levels of academic burnout. (2) Physical exercise was significantly negatively correlated with expressive suppression (β = –0.450, *p* < 0.001); higher levels of physical exercise were associated with a lower tendency toward expressive suppression. (3) Expressive suppression was significantly positively correlated with academic burnout (β = 0.330, *p* < 0.001); higher levels of expressive suppression were associated with more severe academic burnout. (4) Expressive suppression showed a significant partial statistical mediation in the association between physical exercise and academic burnout. The indirect effect was –0.114 [95% CI: (–0.120, –0.097)], accounting for 36.31% of the total effect.

**Conclusion:**

Higher levels of physical exercise were associated with lower academic burnout among middle school students. This association was partially mediated by lower levels of expressive suppression. These findings provide preliminary evidence that school-based physical activity may be relevant to students’ academic adjustment and emotion regulation, but longitudinal and intervention studies are needed before drawing causal or policy-level conclusions.

## 1 Introduction

Academic burnout is a persistent negative psychological state related to learning activities and has become an important concern in adolescent mental health (Gao, 2023). It is typically characterized by emotional exhaustion, academic cynicism, and reduced personal accomplishment (Rosales-Ricardo and Ferreira, 2022). Academic burnout may impair students’ academic performance, mental health, social adaptation, and long-term development (Fu et al., 2023). Adolescents with persistent academic burnout are also more likely to experience anxiety, depression, school alienation, and maladaptive coping styles (Simoës-Perlant et al., 2023; Simoës-Perlant, 2025). Therefore, academic burnout is an important issue for both public health and educational practice.

Physical exercise is a highly accessible and low-cost behavior that has been associated with adolescent mental health (Dhachpramuk et al., 2024; González-Hernández et al., 2019). Prior studies have consistently shown that students who engage in more physical activity tend to report lower levels of academic burnout (Chen et al., 2022; Du et al., 2025; Tong et al., 2023). However, the psychological pathways linking physical exercise to academic burnout remain insufficiently understood. Existing studies have mainly examined positive resource-based mechanisms, such as self-efficacy (Chen et al., 2025), psychological resilience (Liu et al., 2026), and social support (Wang and Jiang, 2025). Less attention has been paid to whether physical exercise is associated with lower use of maladaptive emotion regulation strategies, such as expressive suppression.

Expressive suppression, an important dimension of emotion regulation, refers to an individual’s conscious inhibition of outward emotional expression during an emotional experience (Wu et al., 2025). Psychological research indicates that long-term or habitual use of expressive suppression strategies is often accompanied by higher levels of psychological burden, hindered emotional processing, and increased susceptibility to burnout-related symptoms (Wu et al., 2025). For adolescents, frequent suppression of emotional expression may lead to the accumulation of stress, thereby exacerbating emotional exhaustion and weakening their engagement in learning tasks (Chacón-Cuberos et al., 2019). Recent studies suggest that physical exercise may play a key role in shaping adolescents’ emotion regulation styles (Al-Wardat et al., 2024). Regular physical activity has been found to reduce individuals’ reliance on maladaptive strategies like expressive suppression, potentially due to the facilitation of emotional release during exercise, enhanced interoceptive awareness, and the cultivation of adaptive coping styles (Potoczny et al., 2025). Although considerable theory and empirical evidence point to this possibility, systematic validation of whether expressive suppression statistically mediates the association between physical exercise and academic burnout, specifically among Chinese middle school students, is still lacking.

**FIGURE 1:**
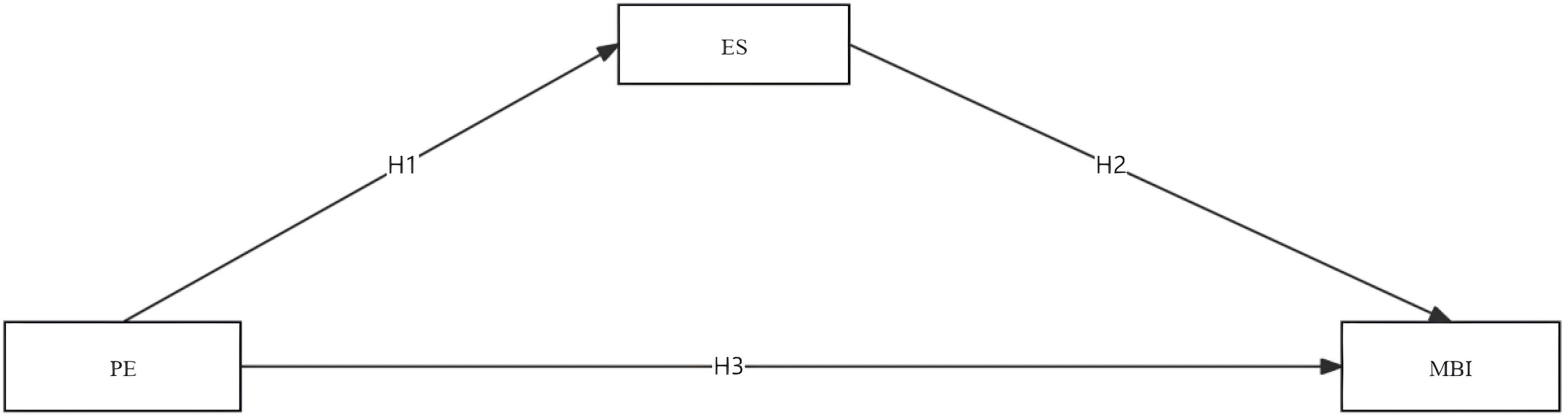
Hypothesized mediation model.

To address this gap, the present study examined the association between physical exercise and academic burnout among Chinese middle school students, with a focus on the mediating role of expressive suppression. The study aimed to clarify a possible psychological pathway linking physical exercise to academic burnout rather than to establish causality. To guide readers through the theoretical logic of the study, To guide readers through the theoretical logic of the study, we present the conceptual mediation model in the Introduction. As shown in Figure 1, physical exercise was hypothesized to be negatively associated with academic burnout both directly and indirectly through lower expressive suppression. The findings may provide preliminary evidence for future school-based research on the role of physical activity in adolescent mental health and academic adjustment.

### 1.1 The relationship between physical exercise and academic burnout

As noted above, academic burnout is characterized by emotional exhaustion, academic cynicism, and reduced personal accomplishment. Given its negative implications for students’ learning and psychological adjustment, identifying feasible factors associated with lower academic burnout is important (Wang et al., 2024). Physical exercise has received increasing attention as one such factor because it is accessible, low cost, and closely related to adolescent mental health (Deng et al., 2025).

Regarding the relationship between physical exercise and academic burnout, existing research provides relatively consistent evidence. Theoretically, Conservation of Resources (COR) theory offers a robust framework for explaining this negative association. This theory posits that individuals are motivated to acquire and protect their valuable resources, such as time, energy, and emotion. When facing continuous academic pressure, students’ psychological resources are depleted; without timely replenishment, resource loss occurs, subsequently leading to burnout (Guo, 2025). Physical exercise can be viewed as a potential means of resource acquisition and restoration. Regular physical activity may improve mood and reduce stress by promoting neurophysiological responses related to positive affect (Silber and Garn, 2025). It may also provide opportunities for goal achievement, skill development, and peer interaction, thereby strengthening self-efficacy and resilience (Chen et al., 2025; Cui and Zhou, 2025). These resources may help students cope with academic demands and may be associated with a lower risk of burnout.

Numerous empirical studies support this theoretical reasoning. Whether targeting university students or adolescents, cross-sectional studies generally find that individuals with higher participation in physical exercise score significantly lower on overall academic burnout and its dimensions (emotional exhaustion, academic cynicism, reduced accomplishment) compared to those lacking exercise (Grace and Moodley, 2024; Ren et al., 2026). For example, Chen et al. (2022) found that physical activity levels among university students were significantly and negatively associated with academic burnout, and this association was partially mediated by self-efficacy and psychological resilience. A recent study by Du et al. (2025) further reported that physical exercise was indirectly associated with lower academic burnout among adolescents through internet addiction and self-control. These studies suggest that physical exercise may be an important external resource associated with students’ coping with academic stress and mental health. Based on this, the present study proposes the following hypothesis:

Research Hypothesis:

*H1:* Physical exercise is significantly negatively correlated with academic burnout in adolescents.

### 1.2 The relationship between physical exercise and expressive suppression

Emotion regulation refers to the processes through which individuals monitor and modify their emotional experiences and expressions (Leunda et al., 2024). According to Gross’s model, expressive suppression is a response-focused strategy that involves inhibiting emotional expression after an emotional response has been generated (Ji et al., 2024). It is often considered maladaptive when used habitually.

While expressive suppression can control overt emotional behavior in the short term, it is a cognitively demanding and inefficient strategy in the long run. It merely masks the external expression of emotion without genuinely alleviating the internal emotional experience. This can paradoxically lead to increased physiological arousal and consume significant cognitive resources, creating a psychological burden (Xu et al., 2026). Chronic, habitual use of expressive suppression hinders emotional processing, leading to an accumulation of negative emotions and ultimately harming mental health (Luoma and Chwyl, 2022).

Recent research suggests that physical exercise may be associated with individuals’ choice of emotion regulation strategies (Vega-Díaz et al., 2025). Firstly, physical activity may provide a natural and socially accepted outlet for emotional release. Whether through high-intensity exercise to vent tension and anger or through calming activities to soothe anxiety, individuals can process and release accumulated emotions non-verbally, reducing the need for suppression (Sánchez-Núñez et al., 2023). Secondly, regular exercise, particularly activities emphasizing the mind-body connection like yoga or Tai Chi, may enhance interoceptive awareness—the ability to perceive internal bodily signals such as heartbeat, breathing, and muscle tension (Wang et al., 2025). This heightened self-awareness helps individuals recognize their emotional changes earlier and more acutely, potentially leading them toward more adaptive regulation strategies rather than resorting to suppression after emotions have escalated (Peng et al., 2025). Thirdly, participating in physical activities, especially team sports, increases positive social interactions and emotional communication, providing individuals with safe contexts to express emotions and receive support, fostering more open and adaptive emotional coping styles (Stefanica et al., 2025).

Research by Al-Wardat et al. (2024) found that physical activity levels were negatively correlated with difficulties in emotion regulation; regularly active individuals demonstrated greater flexibility in emotion regulation. Potoczny et al. (2025) also reported that regular physical activity was associated with higher life satisfaction through self-control and emotion regulation abilities. This evidence suggests that physical exercise may be related to lower reliance on maladaptive strategies such as expressive suppression and to more adaptive emotion regulation patterns. However, this relationship lacks direct empirical testing in the Chinese middle school student population. Therefore, this study proposes the following hypothesis:

*H2:* Physical exercise is significantly negatively correlated with the level of expressive suppression in adolescents.

### 1.3 The relationship between expressive suppression and academic burnout

Expressive suppression and emotional exhaustion are related but distinct constructs. Expressive suppression is a response-focused emotion regulation strategy involving the inhibition of emotional expression. In contrast, emotional exhaustion is a symptom dimension of academic burnout that reflects depleted psychological and emotional resources in academic contexts. Expressive suppression, as a maladaptive emotion regulation strategy, is considered an important correlate of negative psychological outcomes such as burnout. When adolescents frequently employ expressive suppression to cope with daily stress and setbacks, they may experience greater psychological burden (Zhang et al., 2019).

Expressive suppression is a cognitively demanding psychological process. It requires individuals to monitor their emotional state and behavior and to use cognitive resources to inhibit emotional expression. This process may be associated with greater cognitive and emotional resource consumption (Chen et al., 2024). For middle school students facing heavy academic pressure, such additional resource consumption may be linked to greater vulnerability to emotional exhaustion (Gao, 2023).

Expressive suppression may limit the social sharing of emotions and access to interpersonal support. Emotions typically serve social functions; expressing emotions to others may elicit comfort, understanding, and practical help (Yao and Xu, 2024). When adolescents habitually suppress their emotions, especially when facing academic setbacks or interpersonal tensions, they may have fewer opportunities to seek social support (Cameron and Overall, 2018). Unresolved negative emotions may accumulate internally and be associated with a persistent psychological burden. Over time, this state may be linked to lower enthusiasm for learning, more alienated academic attitudes, academic cynicism, and reduced personal accomplishment (Liu et al., 2024).

Research by Chacón-Cuberos et al. (2019) directly explored the relationship between emotion regulation and school burnout, finding that the use of emotion regulation strategies, particularly dimensions related to emotional processing and expression, was significantly correlated with students’ levels of school burnout. Individuals experiencing difficulties with emotion regulation were more likely to exhibit burnout symptoms (Chacón-Cuberos et al., 2019). In summary, a high level of expressive suppression may constitute an important psychological correlate of academic burnout in middle school students. Therefore, this study proposes the following hypothesis:

*H3:* Expressive suppression is significantly positively correlated with academic burnout in adolescents.

In summary, previous studies suggest that physical exercise is negatively associated with academic burnout and expressive suppression, whereas expressive suppression is positively associated with academic burnout. However, the mediating role of expressive suppression in the association between physical exercise and academic burnout has not been directly tested among Chinese middle school students. Therefore, this study examined this mediation model, as shown in Figure 1.

## 2 Materials and methods

### 2.1 Research objects

This study employed a random cluster sampling method. From October 2025 to January 2026, surveys were conducted in various regions: Guangzhou and Heyuan (Guangdong Province), Chengdu and Leshan (Sichuan Province), Hangzhou and Lishui (Zhejiang Province), Beijing, Zhengzhou and Luoyang (Henan Province), and Sanya and Haikou (Hainan Province). Prior to the survey, researchers at the survey sites were trained. This questionnaire is a self-report scale, which may be subject to social desirability and common method bias. The questionnaire is completed entirely anonymously. Participants were informed that there were no right or wrong answers and were encouraged to answer truthfully. Homeroom teachers entered classrooms to explain the study, obtain informed consent from the participants and verbal consent from their guardians. Participants were informed they could terminate the questionnaire completion at any time, and psychological counselors were available in classrooms for support. Questionnaires were completed within 10 min, with homeroom teachers present to explain items if needed, ensuring orderly participation. A total of 4,320 questionnaires were distributed. After collection and cleaning, 534 questionnaires were excluded due to incompleteness, homogeneity, or invalidity, resulting in 3,786 valid questionnaires. Prior to the study, ethical approval was obtained from the Ethics Committee, a subsidiary branch of the Academic Committee of Leshan Normal University (Approval No: LSNU: 1037-26-01RO).

### 2.2 Research instruments

#### 2.2.1 Physical activity rating scale

This study used the Physical Activity Rating Scale developed by Chinese scholar Liang Deqing to measure adolescents’ participation in daily physical exercise. This scale has been used in Chinese adolescent populations and has demonstrated good reliability and validity (Guo et al., 2025). The scale comprises three items assessing exercise frequency, exercise intensity, and exercise duration over the past week. Each item was rated using a 5-point Likert-type scoring format, with higher scores indicating a higher level of the corresponding dimension of physical exercise. In the present SEM analysis, the three items were used as observed indicators of the latent variable “physical exercise,” rather than being combined into a single observed total score. Preliminary testing with a small sample in this study yielded a Cronbach’s alpha coefficient of 0.81, indicating good internal consistency.

#### 2.2.2 Expressive suppression scale

This study used the expressive suppression dimension from the Emotion Regulation Questionnaire developed by Paiča et al. (2020). This scale has demonstrated high reliability and validity in Chinese participant samples in previous studies. The expressive suppression dimension consists of 4 observed items, scored on a Likert 7-point scale. Higher total scores indicate a greater degree of expressive suppression. In the preliminary testing phase of this study, the internal consistency Cronbach’s alpha coefficient for expressive suppression was 0.79, indicating good reliability and validity.

#### 2.2.3 Academic burnout scale

This study used the Adolescent Academic Burnout Scale developed by He et al. (2022) a Chinese version adapted for the local context. It is primarily used to assess burnout, lethargy, distractibility, and lack of focus during the learning process among adolescents. The scale consists of 16 observed items, scored on a Likert 5-point scale. Higher scores indicate a greater degree of academic burnout. In the preliminary testing phase of this study, the internal consistency Cronbach’s alpha coefficient was 0.82, indicating good reliability and validity.

### 2.3 Statistical analysis

Data were analyzed using SPSS 26.0 and AMOS 24.0. SPSS 26.0 was used for descriptive statistics, Pearson correlation analysis, and collinearity diagnostics. Collinearity was assessed using variance inflation factors and tolerance values. AMOS 24.0 was used to estimate the structural equation model using maximum likelihood estimation. Model fit was evaluated using standard fit indices, including χ2/df, CFI, TLI, RMSEA, and SRMR. To maintain consistency in the mediation analysis, the direct, indirect, and total effects were estimated within the SEM framework in AMOS using 5,000 bootstrap samples and bias-corrected 95% confidence intervals. Both unstandardized and standardized coefficients were reported where appropriate. Gender, age, and grade were included as covariates by specifying paths from these variables to expressive suppression and academic burnout in the SEM.

## 3 Results

### 3.1 Common method bias and collinearity diagnostics

As this study primarily used questionnaires, there was a potential risk of systematic error due to the common method. Harman’s single-factor test and the comparison of competing models were employed (Lin et al., 2025). Exploratory factor analysis revealed that the largest factor extracted accounted for 26.3% of the variance, well below the recommended threshold of 40%. Furthermore, comparing competing models showed that the fit indices for the single-factor model were significantly worse than those for the multi-factor model. These results suggest that common method bias was not severe, although it cannot be fully ruled out. Subsequently, collinearity diagnostics were performed. All variance inflation factors (VIF) were < 2, and tolerance coefficients were all greater than the 0.2 threshold. Overall, these results suggest that collinearity among the variables is not a significant issue in this study. Details are shown in Table 1.

**TABLE 1:** Collinearity diagnostics for the regression model.

	Unstandardized coefficients		Standardized coefficients	*T*	*P*	Collinearity statistics	
	B	Std. error	Beta			VIF	Tolerance
(Constant)	1.417	0.257	−	5.504	<0.001	−	−
Gender	–0.05	0.028	–0.027	–1.785	0.074	1.030	0.971
Grade	0.036	0.019	0.032	1.929	0.054	1.284	0.779
Age	0.105	0.018	0.098**	5.815	<0.001	1.298	0.77
PE	–0.195	0.017	–0.181**	–11.266	<0.001	1.182	0.846
ES	0.45	0.025	0.282**	17.638	<0.001	1.168	0.856
*R* ^2^	0.171						
Adj. *R*^2^	0.170						
*F*	*F*(5, 3,780) = 155.885, *p* < 0.001						
D-W value	1.875						

B = Unstandardized regression coefficient, Std. Error = Standard error of the coefficient, Beta = Standardized regression coefficient; *t* = *t*-test statistic for coefficient significance; p = significance probability, **p < 0.01; VIF = Variance Inflation Factor, Tolerance = 1/VIF; *R*^2^ = coefficient of determination; Adj. *R*^2^ = adjusted coefficient of determination; F = F-statistic for overall model significance; D-W Value = Durbin–Watson statistic for autocorrelation of residuals. Typically, VIF < 10 and Tolerance > 0.2 indicate no serious collinearity issues.

### 3.2 Descriptive statistics and correlation coefficients

Table 2 shows the demographic distribution of the participants. Regarding gender, there were 1,914 males (50.55%) and 1,872 females (49.45%). The age distribution was primarily concentrated in the 13–15-year range, with students aged 13, 14, and 15 accounting for 34.36, 32.99, and 29.40% of the sample, respectively, collectively representing over 96% of the participants. Grade distribution was relatively balanced, with students in Grades 7, 8, and 9 comprising 34.52, 33.78, and 31.70% of the sample, respectively. Overall, the demographic distribution of the sample was relatively balanced.

**TABLE 2:** Demographic information.

Variable	Category	Frequency	Percentage (%)	Cumulative percentage (%)
Gender	Male	1,914	50.55	50.55
	Female	1,872	49.45	100
Age	12 years	86	2.27	2.27
	13 years	1,301	34.36	36.63
	14 years	1,249	32.99	69.62
	15 years	1,113	29.40	99.02
	16 years	37	0.98	100.00
Grade	Grade 7	1,307	34.52	34.52
	Grade 8	1,279	33.78	68.30
	Grade 9	1,200	31.70	100.00
Total			3,786	100.00

Table 3 shows that the three dimensions of physical exercise were significantly positively correlated with each other (*r* = 0.595–0.608, *p* < 0.01), indicating high internal consistency among the variables. Each dimension of physical exercise was significantly negatively correlated with each observed item of expressive suppression (*r* = –0.094 to –0.308, *p* < 0.01), suggesting that higher levels of physical exercise are associated with a lower tendency to use expressive suppression. Each dimension of physical exercise was significantly negatively correlated with the dimensions of academic burnout, namely emotional exhaustion, academic cynicism, and reduced personal accomplishment (*r* = –0.233 to –0.274, *p* < 0.01), indicating that higher levels of physical exercise were associated with lower levels of academic burnout. Furthermore, ES1, ES2, and ES3 were moderately and positively correlated with each other (*r* = 0.544–0.621, *p* < 0.01). However, ES4 showed near-zero correlations with ES1–ES3, including a slight negative correlation with ES1. At the same time, ES4 showed weak positive correlations with the three academic burnout dimensions. This atypical item-level pattern suggests that ES4 may function differently from the other expressive suppression items in the present sample. Therefore, this issue was further considered in the measurement-model interpretation and sensitivity analysis.

**TABLE 3:** Means, standard deviations, and correlations among observed variables.

	M	SD	PE1	PE2	PE3	ES1	ES2	ES3	ES4	EE	CD	RPA
PE1	3.870	0.988	1	1	1	1	1	1	1	1	1	1
PE2	4.050	0.963	0.603**									
PE3	3.679	1.105	0.608**	0.595**								
ES1	0.644	0.825	–0.298**	–0.276**	–0.308**							
ES2	0.432	0.730	–0.240**	–0.242**	–0.255**	0.544**						
ES3	0.444	0.781	–0.274**	–0.254**	–0.295**	0.621**	0.558**					
ES4	1.487	1.180	–0.094**	–0.118**	–0.095**	0.522**	0.487**	0.045**				
EE	2.408	0.995	–0.243**	–0.274**	–0.263**	0.341**	0.249**	0.282**	0.095**			
CD	2.448	1.001	–0.233**	–0.243**	–0.253**	0.318**	0.234**	0.269**	0.094**	0.771**		
RPA	2.516	1.087	–0.237**	–0.247**	–0.262**	0.379**	0.260**	0.321**	0.082**	0.694**	0.693**	

M, Mean; SD, Standard Deviation. PE1/PE2/PE3 represent exercise frequency, intensity, and duration, respectively. ES1/ES2/ES3/ES4 represent observed items for expressive suppression. EE, CD, and RPA represent emotional exhaustion, academic cynicism, and reduced personal accomplishment, respectively. *p < 0.05, **p < 0.01, ***p < 0.001.

### 3.3 Mediation effect test

Before testing the structural paths, the overall fit of the structural equation model was evaluated using standard fit indices. As shown in Table 4, the model showed acceptable to good fit to the data, with CFI and TLI above 0.90 and RMSEA and SRMR below 0.08. Although the χ^2^/df value was relatively high, this may be partly attributable to the large sample size. Overall, the fit indices indicated that the proposed model adequately represented the observed data.

**TABLE 4:** Model fit indices of the structural equation model.

Fit index	Value	Recommended	
		Criterion	Interpretation
χ^2^	156.420	–	–
df	35	–	–
χ^2^/df	4.469	<5.00	Acceptable
CFI	0.972	>0.900	Good fit
TLI	0.953	>0.900	Good fit
RMSEA	0.058	<0.080	Acceptable
SRMR	0.038	<0.080	Good fit

χ^2^, chi-square statistic; df, degrees of freedom; CFI, comparative fit index; TLI, Tucker–Lewis index; RMSEA, root mean square error of approximation; SRMR, standardized root mean square residual.

Although the standardized factor loading of ES4 was relatively low, ES4 was retained because it is an item from the original expressive suppression scale and its retention helps preserve the structural integrity of the measurement instrument and maintain comparability with previous studies. To examine whether this low loading affected the substantive findings, a sensitivity analysis was conducted. The results showed that the direction and statistical significance of the main structural paths and the mediation effect remained consistent, indicating that the inclusion of ES4 did not materially alter the main conclusions of the model.

**FIGURE 2:**
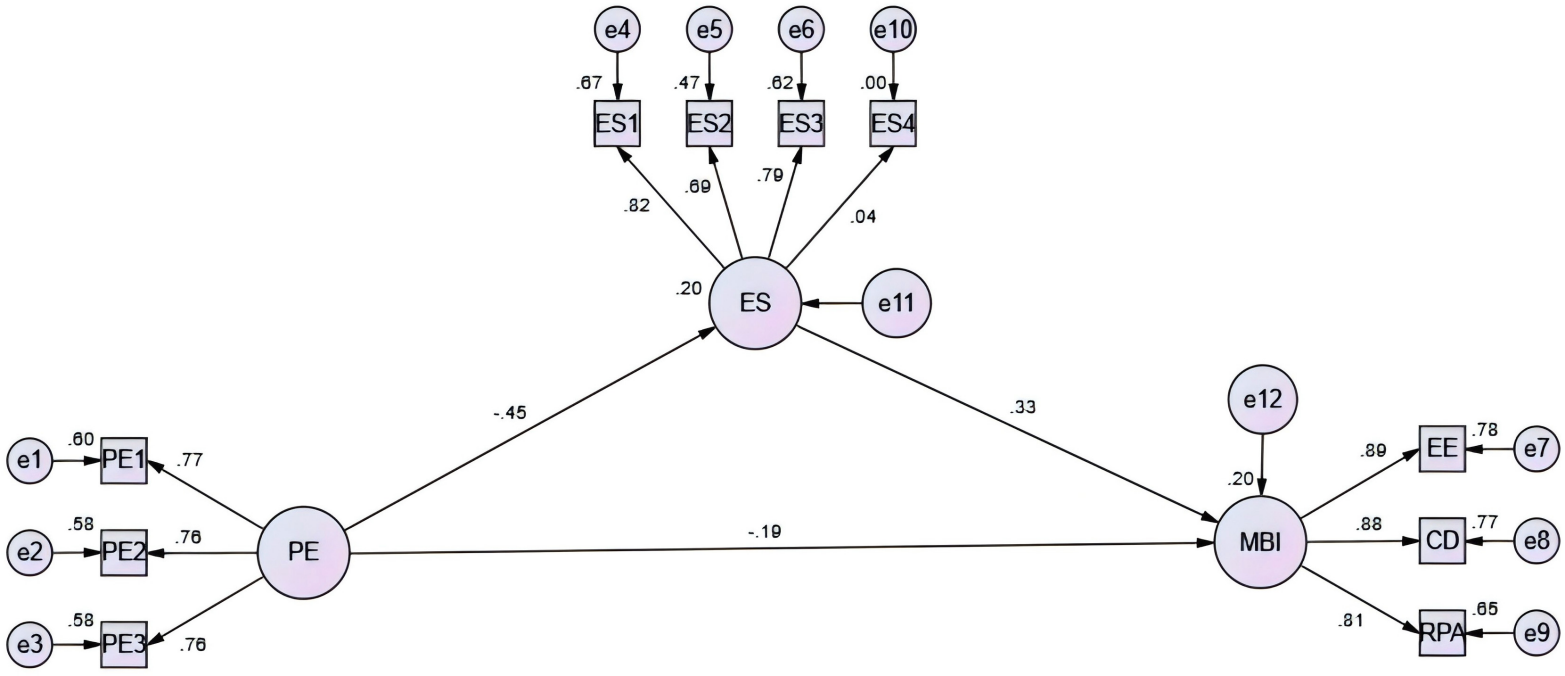
Structural equation model with standardized path coefficients.

The structural model was examined using AMOS 24.0 to test the associations among physical exercise, expressive suppression, and academic burnout. Gender, age, and grade were included as covariates in the tested model, although these covariate paths are not displayed in Figure 2 to maintain visual clarity. The results are presented in Figure 2 and Table 5. Physical exercise was significantly and negatively associated with expressive suppression (β = –0.450, *p* < 0.001), indicating that students with higher levels of physical exercise tended to report lower levels of expressive suppression. Physical exercise was also significantly and negatively associated with academic burnout (β = –0.192, *p* < 0.001). In addition, expressive suppression was significantly and positively associated with academic burnout (β = 0.330, *p* < 0.001), suggesting that students with higher levels of expressive suppression tended to report more severe academic burnout. These results support the hypothesized path relationships among physical exercise, expressive suppression, and academic burnout, thereby supporting H1, H2, and H3.

**TABLE 5:** Structural path coefficients and measurement loadings.

Path			Unstandardized coefficient	S.E.	AVE	C.R.	Standardized coefficient
ES	<—	PE	–0.397	0.018		–22.044	–0.450***
MBI	<—	PE	–0.221	0.024		–9.228	–0.192***
MBI	<—	ES	0.431	0.028		15.636	0.330***
PE1	< —	PE	1.000		0.588		0.774
PE2	<—		0.961	0.023		41.310	0.764***
PE3	<—		1.102	0.027		41.277	0.763***
ES1	<—	ES	1.000		0.441		0.817
ES2	<—		0.744	0.019		39.689	0.687***
ES3	<—		0.914	0.021		43.496	0.789***
ES4	<—		0.078	0.031		2.504	0.045*
EE	<—	MBI	1.000		0.735		0.886
CD	<—		0.997	0.015		66.730	0.877***
RPA	<—		0.996	0.016		60.445	0.807***

PE, physical exercise; ES, expressive suppression; MBI, academic burnout. PE1 = exercise frequency, PE2 = exercise intensity, PE3 = exercise duration. ES1–ES4 = observed items of expressive suppression. EE, emotional exhaustion; CD, academic cynicism; RPA, reduced personal accomplishment. SE, standard error; AVE, average variance extracted, reported at the latent-construct level where applicable; CR, critical ratio, calculated as the unstandardized coefficient divided by its standard error. *p < 0.05, **p < 0.01, ***p < 0.001.

To maintain consistency with the SEM-based path analysis, the mediating role of expressive suppression was further tested within the same AMOS SEM framework using 5,000 bootstrap samples and bias-corrected 95% confidence intervals. In response to the potential influence of demographic differences, gender, age, and grade were included as covariates in the mediation model. As shown in Table 6, after controlling for these demographic covariates, the indirect effect of physical exercise on academic burnout through expressive suppression remained significant [Effect = –0.114, Boot SE = 0.007, 95% CI (–0.120, –0.097), *p* < 0.001]. The confidence interval did not include zero, indicating a significant statistical mediation effect. Specifically, higher levels of physical exercise were associated with lower levels of expressive suppression, which in turn were associated with lower levels of academic burnout. These results support the proposed mediation model.

**TABLE 6:** SEM-based bootstrap estimates of direct, indirect, and total effects.

Path	Effect	95% *CI*		Boot SE	*z/t*-value	*P-*value	Effect ratio
		Lower	Upper				
Indirect effect: PE → ES → MBI	–0.114	–0.120	–0.097	0.007	–17.493	<0.001	36.31%
Path a: PE → ES	–0.250	–0.270	–0.230	0.010	–24.584	<0.001	/
Path b: ES → MBI	0.456	0.406	0.507	0.026	17.829	<0.001	/
Direct effect: PE → MBI	–0.200	–0.234	–0.166	0.017	–11.585	<0.001	63.69%
Total effect: PE → MBI	–0.314	–0.347	–0.281	0.017	–18.828	<0.001	/

PE, Physical Exercise; ES, Expressive Suppression; MBI, Academic Burnout. Gender, age, and grade were controlled as demographic covariates in the SEM-based bootstrap mediation model. For clarity, covariate paths are not shown in Figure 2. * indicates *p* < 0.05, ** indicates *p* < 0.01, *** indicates *p* < 0.001. Effect values are effect sizes estimated by the Bootstrap method. 95% CI = Bias-corrected Bootstrap confidence interval; interval not containing 0 indicates a significant effect. Boot SE = Bootstrap standard error. z/t-value = statistic for significance test. Effect ratio = percentage of indirect effect relative to total effect.

In terms of effect decomposition, the direct effect of physical exercise on academic burnout remained significant [Effect = –0.200, Boot SE = 0.017, 95% CI (–0.234, –0.166), *p* < 0.001], and the total effect was also significant [Effect = –0.314, Boot SE = 0.017, 95% CI (–0.347, –0.281), *p* < 0.001]. The indirect effect accounted for 36.31% of the total effect, whereas the direct effect accounted for 63.69%. These findings indicate a partial statistical mediation pattern. Specifically, physical exercise was directly associated with lower academic burnout and was also indirectly associated with lower academic burnout through lower levels of expressive suppression. Overall, the results from the consistent SEM-based bootstrap approach support the proposed partial mediation model. However, because the study used a cross-sectional design, these findings should be interpreted as statistical associations rather than causal effects.

## 4 Discussion

### 4.1 The association between physical exercise and academic burnout

This study found a significant negative association between physical exercise and academic burnout among Chinese middle school students, supporting H1. Students with higher levels of physical exercise tended to report lower levels of emotional exhaustion, academic cynicism, and reduced personal accomplishment. This finding is consistent with previous studies (Grace and Moodley, 2024; Ren et al., 2026) and extends the evidence to a large sample of Chinese middle school students.

From a theoretical perspective, this finding can be interpreted using Conservation of Resources (COR) theory. Academic burnout may be related to the continuous depletion of students’ emotional and cognitive resources under prolonged academic stress, especially when resources are not sufficiently replenished (Cuevas-Caravaca et al., 2024). The results of this study suggest that physical exercise may be understood as a potential form of “resource investment” associated with lower levels of academic burnout. Physiologically, regular physical exercise may be linked to stress regulation and positive affective responses (González-Hernández et al., 2019). Psychologically, physical activities may be associated with autonomy, competence, and relatedness by providing opportunities for goal achievement, skill improvement, and peer interaction (Dang, 2021). These positive psychological experiences may serve as psychological resources associated with better academic adjustment and lower burnout risk (Luo and Peng, 2026).

Furthermore, physical exercise may be indirectly associated with adolescents’ ability to cope with academic stress through psychological resilience and self-efficacy (Li et al., 2024). Students who maintain long-term exercise habits often demonstrate greater persistence and a problem-solving orientation when facing challenges. This positive coping style may also be relevant to the academic domain and may be associated with lower helplessness and disengagement when students confront heavy coursework (Qiu et al., 2025). Therefore, physical exercise may be considered not only a physical activity but also a potential correlate of positive psychological qualities and stress buffering (Zhao et al., 2022).

It is noteworthy that the statistical direct effect accounted for 63.69% of the total effect. This indicates that expressive suppression explained only part of the association between physical exercise and academic burnout. This finding suggests that physical activity may be a potentially relevant correlate of students’ psychological adjustment within the school context. However, because the present study was cross-sectional, physical exercise should be viewed as one possible component for further investigation in broader educational and mental health promotion efforts, rather than as an established intervention strategy for reducing academic burnout (Bai et al., 2022).

### 4.2 The Association between physical exercise and expressive suppression

This study found a significant negative association between physical exercise and expressive suppression. Among the three hypothesized paths, this association showed the largest standardized coefficient. This suggests that physical exercise may be closely related to adolescents’ emotion regulation styles. Although ES4 showed a weak factor loading, the sensitivity analysis indicated that this measurement issue did not alter the substantive interpretation of the association between physical exercise and expressive suppression.

From an emotion regulation perspective, expressive suppression, as a response-focused strategy, is characterized by the individual’s conscious control over the outward expression of emotion. While this control can maintain appropriate emotional displays in the short term, it requires continuous investment of cognitive resources and fails to resolve the internal emotional experience (Zhang et al., 2025). The strong negative association found in this study between physical exercise and expressive suppression may be related to the fact that physical activity may provide a context for emotional processing (Tao et al., 2025). During physical activity, physiological activation may increase, which may be associated with emotional release and transformation (Long et al., 2021). In other words, students who engage in more physical exercise may be less likely to rely on cognitive suppression strategies when processing emotions (Bernstein and Mcnally, 2018).

Another possible explanation is that students who regularly participate in physical activities may be developing a more automatic mode of emotional coping (Leunda et al., 2024). Unlike cognitive regulation strategies that require conscious participation, this mode is closer to procedural knowledge. The individual does not need to deliberately decide how to regulate emotions; instead, emotional transformation occurs naturally through the process of physical activity (Tao et al., 2023). From a developmental perspective on emotion regulation, this embodied regulation style may be more suitable for adolescents (Xu et al., 2025). Since the prefrontal cortex function in adolescents is not yet fully developed, their cognitive control over emotions is limited. Regulation at the physical level, which relies less on cognitive resources, may be more aligned with the characteristics of their developmental stage (Mu and Lu, 2025).

This study extends research on the psychological correlates of physical exercise from an emotion regulation perspective, suggesting that physical activity may be closely associated with adolescents’ emotion regulation strategies.

### 4.3 The mediating role of expressive suppression

This study found that expressive suppression partially statistically mediated the association between physical exercise and academic burnout. This result suggests a possible pathway in which higher levels of physical exercise are associated with lower reliance on expressive suppression, which in turn is associated with lower academic burnout.

The theoretical significance of this finding lies in suggesting that researchers should pay attention to possible risk-related pathways. Previous research on the mediating mechanisms between physical exercise and academic burnout has largely focused on positive psychological resources, such as self-efficacy (Mu et al., 2024) and psychological resilience (Zhang et al., 2024). These can be summarized as “resource gain” pathways. The present study adds that lower use of maladaptive emotion regulation strategies may also be involved in the association between physical exercise and academic burnout. This can be considered a possible “risk reduction” pathway. Together, these pathways may provide a more complete picture of how physical exercise is linked to academic burnout.

Comparing the effect sizes, the association between physical exercise and expressive suppression was notably stronger than the association between expressive suppression and academic burnout. This pattern suggests that expressive suppression may function as a relatively proximal statistical mediator in the proposed model. However, because the data are cross-sectional, the temporal ordering among these variables cannot be confirmed. This interpretation requires further validation through longitudinal studies.

Furthermore, the presence of a partial statistical mediation effect indicates that expressive suppression explains only part of the association between physical exercise and academic burnout. Besides the emotion regulation pathway, other factors, such as sleep quality (Roig et al., 2022), social interaction (Surkalim et al., 2024), and academic self-efficacy (Gao et al., 2025), may also be involved. Future research could examine the independent contributions and interactions of these variables within more integrated models to develop a more comprehensive understanding of the correlates linking physical exercise and academic burnout.

## 5 Conclusion and implications

### 5.1 Conclusion

(1) Physical exercise is significantly negatively correlated with academic burnout in middle school students. Higher levels of physical exercise are associated with lower levels of emotional exhaustion, academic cynicism, and reduced personal accomplishment.

(2) Physical exercise is significantly negatively correlated with expressive suppression. Higher levels of physical exercise are associated with a lower tendency to use expressive suppression.

(3) Expressive suppression is significantly positively correlated with academic burnout and partially mediates the relationship between physical exercise and academic burnout. The indirect effect accounts for 36.31% of the total effect.

### 5.2 Implications

Because this study used a cross-sectional design, the practical implications should be interpreted as preliminary and correlational rather than causal.

(1) Schools may consider physical activity as one potentially relevant component of broader student development and mental health promotion efforts. Rather than treating physical exercise as a confirmed intervention for academic burnout, school-based physical activity programs could be further examined as part of integrated approaches to supporting students’ academic adjustment and emotional wellbeing.

(2) Educators and school administrators may also consider the potential association between physical activity and emotion regulation. Physical education classes and extracurricular sports activities may provide contexts for emotional expression and social interaction, which could be relevant to students’ use of emotion regulation strategies. However, these possibilities require further verification through longitudinal and intervention studies.

(3) Given the academic pressure faced by many middle school students, schools may explore diverse and flexibly accessible physical activity opportunities that are responsive to students’ interests and needs. Such efforts should be understood as preliminary practice-oriented considerations based on correlational evidence, rather than definitive recommendations for reducing academic burnout.

## 6 Limitations and future directions

This study has several limitations. First, the cross-sectional design precludes causal inference. Although the model was based on Conservation of Resources theory and emotion regulation theory, the findings should be interpreted as associations rather than causal effects. Therefore, the practical implications should be viewed as preliminary and hypothesis-generating rather than as evidence for a confirmed school-based intervention strategy. Longitudinal studies and randomized controlled trials are needed to examine temporal and causal pathways among these variables.

Second, all core variables were assessed using self-report questionnaires, which may introduce social desirability bias, recall bias, and common method variance. Although Harman’s single-factor test and competing model comparisons suggested that common method bias was not severe, these procedures cannot completely eliminate this concern. Future studies should incorporate objective indicators of physical activity, such as accelerometers, pedometers, or school-based physical fitness records, and collect multi-informant data from teachers, parents, or peers to improve measurement validity.

Third, although the SEM-based mediation model controlled for basic demographic variables, including gender, age, and grade, other important confounding factors were not measured. These may include socioeconomic status, academic achievement, sleep quality, family educational expectations, school climate, and previous mental health history. These omitted variables may simultaneously influence physical exercise, emotion regulation, and academic burnout. Therefore, the observed associations should be interpreted cautiously, and future studies should include a broader range of covariates to further test the robustness of the proposed model.

Fourth, although this study provided preliminary evidence for the empirical distinction between expressive suppression and academic burnout, potential conceptual overlap should still be acknowledged. Expressive suppression is an emotion regulation strategy, whereas emotional exhaustion is a symptom dimension of academic burnout. However, maladaptive emotion regulation and emotional exhaustion are theoretically related. Future studies should further examine discriminant validity by using alternative measurement tools, testing separate burnout dimensions, and applying longitudinal or multi-method designs.

Fifth, the sample was limited to Chinese middle school students. Cultural norms regarding emotional restraint, family educational expectations, and academic competition may shape the observed associations. Therefore, generalization to other age groups, countries, or educational systems should be made cautiously. Future studies should conduct cross-cultural and cross-stage comparisons.

## Data Availability

The original contributions presented in this study are included in the article/supplementary material, further inquiries can be directed to the corresponding author.

## References

[B1] Al-Wardat M. Salimei C. Alrabbaie H. Etoom M. Khashroom M. Clarke C. et al. (2024). Exploring the links between physical activity, emotional regulation, and mental well-being in Jordanian university students. *J. Clin. Med.* 13:1533. 10.3390/jcm13061533 38541759 PMC10970980

[B2] Bai M. Z. Yao S. J. Ma Q. S. Wang X. Liu C. Guo K. et al. (2022). The relationship between physical exercise and school adaptation of junior students: A chain mediating model. *Front. Psychol.* 13:977663. 10.3389/fpsyg.2022.977663 36186376 PMC9519055

[B3] Bernstein E. E. Mcnally R. J. (2018). Exercise as a buffer against difficulties with emotion regulation: A pathway to emotional wellbeing. *Behav. Res. Therapy* 109 29–36. 10.1016/j.brat.2018.07.010 30081242

[B4] Cameron L. D. Overall N. C. (2018). Suppression and expression as distinct emotion-regulation processes in daily interactions: Longitudinal and meta-analyses. *Emotion* 18 465–480. 10.1037/emo0000334 28569538

[B5] Chacón-Cuberos R. Martínez-Martínez A. García-Garnica M. Pistón-Rodríguez M. Expósito-López J. (2019). The relationship between emotional regulation and school burnout: Structural equation model according to dedication to tutoring. *Int. J. Environ. Res. Public Health* 16:4703. 10.3390/ijerph16234703 31779141 PMC6926892

[B6] Chen C. W. Mccarron G. P. Owen J. E. (2024). The emotional labour of college student activism: An interview-based study. *J. Hum. Rights Pract.* 16 964–980. 10.1093/jhuman/huae025

[B7] Chen J. Liu X. Xie J. Yang Q. Fan Z. Peng D. et al. (2025). Effects of physical activity on academic burnout among rural left-behind children in China: The chain-mediated roles of loneliness and general self-efficacy. *Front. Psychol.* 16:1653243. 10.3389/fpsyg.2025.1653243 41209803 PMC12589049

[B8] Chen K. Liu F. Mou L. Zhao P. Guo L. (2022). How physical exercise impacts academic burnout in college students: The mediating effects of self-efficacy and resilience. *Front. Psychol.* 13:964169. 10.3389/fpsyg.2022.964169 36438387 PMC9691659

[B9] Cuevas-Caravaca E. Sánchez-Romero E. I. Antón-Ruiz J. A. (2024). Academic burnout, personality, and academic variables in university students. *Eur. J. Invest. Health Psychol. Educ.* 14 1561–1571. 10.3390/ejihpe14060103 38921069 PMC11202737

[B10] Cui H. C. Zhou Y. (2025). The impact of physical activity on subjective well-being: The mediating role of exercise identity and the moderating role of health consciousness. *PLoS One* 20:e0313799. 10.1371/journal.pone.0313799 40267053 PMC12017510

[B11] Dang L. (2021). Physical exercises in relieving the current state of depression. *Rev. Bras. Med. Esporte* 27 776–778. 10.1590/1517-8692202127082021_0370

[B12] Deng D. Sun Q. Li H. (2025). The influences of physical exercise on student burnout: Based on the mediating role of psychological resilience. *BMC Psychol.* 13:114. 10.1186/s40359-025-02394-9 39934909 PMC11817967

[B13] Dhachpramuk D. Sonjaipanich S. Theppiban S. In-Iw S. (2024). Exercise, mental well-being and burnout in Thai medical students in 2020–2021: An online cross-sectional survey. *BMC Med. Educ.* 24:837. 10.1186/s12909-024-05843-y 39095768 PMC11297641

[B14] Du J. Dong J. Shi Y. Jiang E. Mo L. Wan B. et al. (2025). The relationship between physical exercise and academic burnout in adolescents: The chain-mediated role of internet addiction and self-control. *Front. Psychol.* 16:1710564. 10.3389/fpsyg.2025.1710564 41394052 PMC12695792

[B15] Fu W. Li Y. Liu Y. Li D. Wang G. Liu Y. et al. (2023). The influence of different physical exercise amounts on learning burnout in adolescents: The mediating effect of self-efficacy. *Front. Psychol.* 14:1089570. 10.3389/fpsyg.2023.1089570 36891208 PMC9986600

[B16] Gao W. Chen J. Tu Z. Li M. (2025). Correlational research on college students’ physical exercise behavior, academic engagement, and self-efficacy. *Front. Psychol.* 16:1428365. 10.3389/fpsyg.2025.1428365 39963677 PMC11830674

[B17] Gao X. (2023). Academic stress and academic burnout in adolescents: A moderated mediating model. *Front. Psychol.* 14:1133706. 10.3389/fpsyg.2023.1133706 37342640 PMC10278958

[B18] González-Hernández J. Gómez-López M. Pérez-Turpin J. A. Muñoz-Villena A. Andreu-Cabrera E. (2019). Perfectly active teenagers. When does physical exercise help psychological well-being in adolescents? *Int. J. Environ. Res. Public Health* 16:4525. 10.3390/ijerph16224525 31731765 PMC6888202

[B19] Grace J. M. Moodley L. (2024). Correlation between physical activity and burnout amongst allied healthcare professionals in eThekwini, South Africa. *Sage Open Med.* 12:20503121241297060. 10.1177/20503121241297060 39526093 PMC11549708

[B20] Guo J. (2025). The dual impact of physical exercise on university students’ mental health: The chain mediating effects of mindfulness and psychological resilience. *Front. Psychol.* 16:1545370. 10.3389/fpsyg.2025.1545370 40297594 PMC12034701

[B21] Guo Y. J. Wei B. H. Chen Z. H. (2025). Intra-individual analysis of the relationship between physical exercise and early internalizing and externalizing problems in adolescents: Evidence from longitudinal tracking and diary methods. *Sports Sci.* 45 39–50.

[B22] He A. M. Wan J. J. Hui Q. P. (2022). The relationship between mobile phone dependence and mental health of adolescents: The mediating role of academic burnout and the moderating role of coping styles. *Psychol. Dev. Educ.* 38 391–398.

[B23] Ji F. Sun Q. Han W. Li Y. Xia X. (2024). How physical exercise reduces problematic mobile phone use in adolescents: The roles of expression suppression, depression, anxiety, and resilience. *Psychol. Res. Behav. Manag.* 17 4369–4382. 10.2147/PRBM.S484089 39722776 PMC11669333

[B24] Leunda I. Jauregui P. Figueras S. (2024). Exercise dependence in endurance sports: Relation to emotional regulation and negative affectivity. *Behav. Psychol.* 32 181–202. 10.51668/bp.8324109n

[B25] Li N. Wang D. Zhao X. Li Z. Zhang L. (2024). The association between physical exercise behavior and psychological resilience of teenagers: An examination of the chain mediating effect. *Sci. Rep.* 14:9372. 10.1038/s41598-024-60038-1 38654069 PMC11039466

[B26] Lin Y. He M. Zhou W. Zhang M. Wang Q. Chen Y. et al. (2025). The relationship between physical exercise and psychological capital in college students: The mediating role of perceived social support and self-control. *BMC Public Health* 25:581. 10.1186/s12889-025-21856-8 39939931 PMC11823263

[B27] Liu T. Peng B. Chen W. Ren Y. Hu H. (2026). Green exercise, nature connectedness, and academic burnout: A psychological study based on Chinese university students. *Front. Psychol.* 16:1735287. 10.3389/fpsyg.2025.1735287 41583780 PMC12823991

[B28] Liu Y. Chai X. Sang B. Zhang S. (2024). Differences in the effect of adolescents’ strategies for expressing academic emotions on academic emotions and peer acceptance in competitive and cooperative situations. *Front. Psychol.* 15:1407885. 10.3389/fpsyg.2024.1407885 39021655 PMC11252489

[B29] Long Z. Liu G. Xiao Z. Gao P. (2021). Improvement of emotional response to negative stimulations with moderate-intensity physical exercise. *Front. Psychol.* 12:656598. 10.3389/fpsyg.2021.656598 34276479 PMC8280290

[B30] Luo L. Peng P. (2026). Sleep procrastination and adolescent subjective well-being: The mediating role of emotional exhaustion and the moderating effect of physical exercise. *Acta Psychol.* 262:106060. 10.1016/j.actpsy.2025.106060 41370898

[B31] Luoma J. B. Chwyl C. (2022). Interpersonal mechanisms for the maintenance of self-criticism: Expressive suppression, emotion expression, and self-concealment. *Curr. Psychol.* 41 4027–4040. 10.1007/s12144-020-00920-z

[B32] Mu F. Z. Lu L. (2025). Effects of physical exercise on negative emotions in adolescents: Sequential mediation through psychological benefits and social self-efficacy. *Prevent. Med. Rep.* 58:103231. 10.1016/j.pmedr.2025.103231 40918689 PMC12410517

[B33] Mu F. Liu J. Lou H. Zhu W. D. Wang Z. C. Li B. (2024). How breaking a sweat affects mood: The mediating role of self-efficacy between physical exercise and emotion regulation ability. *PLoS One* 19:e0303694. 10.1371/journal.pone.0303694 38870188 PMC11175485

[B34] Paiča I. Mārtinsone K. Taube M. (2020). Emotion regulation difficulties in depression. *Proc. Int. Sci. Conf.* 7:145. 10.17770/sie2020vol7.4850

[B35] Peng B. Chen W. Wang H. Yu T. Kong M. A. (2025). A study on the relationship between physical exercise and feelings of inferiority among college students: The chain mediating effect of social support and emotional regulation ability. *Front. Psychol.* 15:1521510. 10.3389/fpsyg.2024.1521510 39850972 PMC11754417

[B36] Potoczny W. Herzog-Krzywoszańska R. Krzywoszański Ł (2025). Regular physical activity and life satisfaction: Unpacking the roles of self-control and emotion regulation. *Appl. Sci.* 15:1878. 10.3390/app15041878 PMC8792757 35095440

[B37] Qiu W. Wang X. Cui H. Ma W. Xiao H. Qu G. et al. (2025). The impact of physical exercise on college students’ physical self-efficacy: The mediating role of psychological resilience. *Behav. Sci.* 15:541. 10.3390/bs15040541 40282162 PMC12024398

[B38] Ren Y. Ai L. Yang Y. Zhu F. (2026). Examining the influence of aerobic exercise on middle school students’ academic engagement: Exploring the role of academic buoyancy. *Int. J. Sport Exerc. Psychol.* 24 45–66. 10.1080/1612197X.2024.2437483

[B39] Roig M. Cristini J. Parwanta Z. Ayotte B. Rodrigues L. de Las Heras B. et al. (2022). Exercising the sleepy-ing brain: Exercise, sleep, and sleep loss on memory. *Exerc. Sport Sci. Rev.* 50 38–48. 10.1249/JES.0000000000000273 34669627

[B40] Rosales-Ricardo Y. Ferreira J. P. (2022). Effects of physical exercise on burnout syndrome in university students. *MEDICC Rev.* 24:36. 10.37757/MR2022.V24.N1.7 35157638

[B41] Sánchez-Núñez M. T. Alfaro Portero G. García-Rubio N. (2023). Mental Health and their relationship with physical activity and emotional regulation strategies in Spanish adolescents. *Ansiedad Estrés* 29 63–70. 10.5093/anyes2023a7

[B42] Silber E. Garn A. (2025). Psychological distress in a sample of predominately white female college students: The role of mindfulness and physical activity. *J. Am. College Health* 73 871–878. 10.1080/07448481.2023.2258412 37773603

[B43] Simoës-Perlant A. (2025). Stress, anxiété, burnout scolaire: Comment vont les adolescents français? *Revue des sciences de l’éducation* 50:1115499ar. French. 10.7202/1115499ar

[B44] Simoës-Perlant A. Barreau M. Vezilier C. (2023). Stress, anxiety, and school burnout post COVID-19: A study of french adolescents. *Mind Brain Educ.* 17 98–106. 10.1111/mbe.12346

[B45] Stefanica V. Macri A. C. Man C. M. Vasile A. (2025). Integrated physical education for mental health: Enhancing emotional regulation, self-esteem, and stress resilience in adolescents through sports, mindfulness, and reflection. *Rev. Romaneasca Pentru Educ. Multidimensionala* 17 295–312. 10.18662/rrem/17.3/1023

[B46] Surkalim D. L. Clare P. J. Eres R. Gebel K. Bauman A. Ding D. (2024). Exercise to socialize? Bidirectional relationships between physical activity and loneliness in middle-aged and older American adults. *Am. J. Epidemiol.* 193 996–1001. 10.1093/aje/kwae001 38319704 PMC11228862

[B47] Tao B. Lu T. Chen H. Yan J. (2023). The relationship between psychological stress and emotional state in Chinese university students during COVID-19: The moderating role of physical exercise. *Healthcare* 11:695. 10.3390/healthcare11050695 36900700 PMC10001233

[B48] Tao B. Lu T. Chen H. Yan J. (2025). The effects of moderate- to high-intensity physical exercise on emotion regulation and subsequent cognitive control in highly psychologically stressed college students. *Healthcare* 13:2100. 10.3390/healthcare13172100 40941453 PMC12428589

[B49] Tong J. Zhang Z. Chen W. He Z. (2023). How physical fitness influences academic burnout in elementary students: An interpersonal perspective. *Curr. Psychol.* 42 5977–5985. 10.1007/s12144-021-01948-5

[B50] Vega-Díaz M. Martinent G. González-García H. (2025). The relationship between motivation profiles for health-oriented physical activity, basic psychological needs and emotional regulation. *J. Health Psychol.* 30 1809–1824. 10.1177/13591053241240981 38527942

[B51] Wang S. Huang Y. Si X. Zhang H. Zhai M. Fan H. et al. (2025). The impact of Tai Chi on emotional regulation efficacy and subjective wellbeing in the elderly and the mediating mechanism. *Front. Psychol.* 16:1550174. 10.3389/fpsyg.2025.1550174 40486896 PMC12141313

[B52] Wang X. C. Zhang M. Wang J. X. (2024). The effect of university students’ academic self-efficacy on academic burnout: The chain mediating role of intrinsic motivation and learning engagement. *J. Psychoeduc. Assess.* 42 798–812. 10.1177/07342829241252863

[B53] Wang X. Jiang Y. (2025). The effect of family support on junior high school students’ engagement in physical education—A moderated chain mediation model. *Front. Psychol.* 16:1606642. 10.3389/fpsyg.2025.1606642 40831488 PMC12358406

[B54] Wu J. Shao Y. Hu J. Zhao X. (2025). The impact of physical exercise on adolescent social anxiety: The serial mediating effects of sports self-efficacy and expressive suppression. *BMC Sports Sci. Med. Rehabil.* 17:57. 10.1186/s13102-025-01107-4 40121514 PMC11929206

[B55] Xu Y. Qi K. Meng S. Dong X. Wang S. Chen D. et al. (2025). The effect of physical activity on resilience of Chinese children: The chain mediating effect of executive function and emotional regulation. *BMC Pediatrics* 25:563. 10.1186/s12887-025-05883-3 40696298 PMC12281779

[B56] Xu Y. Zhang G. Q. Tsai W. (2026). Longitudinal associations between expressive suppression and psychological health: The moderating role of authenticity and the ambivalence over emotion expression. *J. Counseling Psychol.* 73 116–127. 10.1037/cou0000834 41213566

[B57] Yao Y. Xu D. (2024). Unconscious cognitive reappraisal and unconscious expression suppression regulate emotional responses: An ERP study. *Curr. Psychol.* 43 7772–7784. 10.1007/s12144-023-04943-0

[B58] Zhang J. Liu H. Dai W. (2024). Physical exercise and positive coping styles of Chinese college students: Psychological resilience as a mediator. *Soc. Behav. Pers. Int. J.* 52 13098E–13106E. 10.2224/sbp.13098

[B59] Zhang T. Wang Z. Liu G. (2019). Teachers’ caring behavior and problem behaviors in adolescents: The mediating roles of cognitive reappraisal and expressive suppression. *Pers. Individ. Dif.* 142 270–275. 10.1016/j.paid.2018.10.005

[B60] Zhang Y. He J. Ruan H. Yao P. Ma G. (2025). The impact of physical exercise on mental health and the relationship among physical exercise, emotional regulation and suicidal ideation in Chinese medical students. *Front. Psychol.* 16:1609415. 10.3389/fpsyg.2025.1609415 40873524 PMC12379471

[B61] Zhao Z. Zhao S. Wang Q. Zhang Y. Chen C. (2022). Effects of physical exercise on mobile phone addiction in college students: The chain mediation effect of psychological resilience and perceived stress. *Int. J. Environ. Res. Public Health* 19:15679. 10.3390/ijerph192315679 36497752 PMC9738933

